# Research on Fire Source Recognition and Fire Extinguishing Algorithms Based on Multimodal Fusion and Lightweight Model Deployment

**DOI:** 10.3390/s26133988

**Published:** 2026-06-23

**Authors:** Daoshang Zhai, Qianjuan Zhai, Shuo Liu, Xiuyan Liu, Tingting Guo

**Affiliations:** 1 School of Electromechanical and Automotive Engineering, Yantai University, Yantai 264005, China; 202356503332@s.ytu.edu.cn (D.Z.); 202400356059@s.ytu.edu.cn (S.L.); 2 Department of Food Science and Engineering, Qilu University of Technology (Shandong Academy of Sciences), Heze 274009, China; 202495033045@stu.qlu.edu.cn; 3 School of Information Management, Qingdao University of Technology, Qingdao 266525, China; liuxiuyan@qut.edu.cn

**Keywords:** fire detection, multi-modal fusion, lightweight FOMO model, sixth-degree polynomial jet model, embedded systems, closed-loop control

## Abstract

Conventional fire monitoring systems frequently exhibit high false alarm rates, delayed response times, and a lack of closed-loop control capabilities, which severely constrain their deployment in complex real-world environments. To address these issues, this paper proposes an embedded fire detection, tracking, and extinguishing system based on multimodal information fusion and a lightweight neural model. The system follows a “Perception–Decision–Execution–Feedback” closed-loop paradigm and is implemented on a heterogeneous cooperative computing architecture comprising OpenMV4 H7 Plus and STM32F103C8T6 microcontrollers. The perception layer implements a decision-level RGB-infrared fusion mechanism that incorporates a pruned, INT8-quantized lightweight FOMO model, enabling real-time fire detection with an inference latency of 210 ms and a model size of merely 1.8 MB under resource-constrained embedded conditions. The decision layer employs a Bayesian inference-based multimodal fusion framework that effectively suppresses spurious fire interference. The vision-only false detection rate is 15.3%. After infrared fusion verification, the system-level false alarm rate is reduced to 2.0% on the interference test set. In the execution layer, a sixth-degree polynomial jet trajectory model was established and combined with an improved PID–PI dual-loop controller to enable dynamic optimization of spray angle and flow rate in real time. Experimental results demonstrate that the proposed system achieves an average fire recognition accuracy of 95.6% with a false alarm rate as low as 1.4%. Furthermore, it realizes an extinguishing accuracy better than ±5 cm within an effective operating range of 10–60 cm and completes the entire perception-to-extinguishing cycle within 8.5 s under illumination conditions ranging from 50 to 100,000 lux. These results demonstrate the excellent real-time capability, robustness, and energy efficiency of the proposed system, providing a practical and scalable solution for autonomous embedded fire-fighting applications in household, industrial, and warehouse environments.

## 1. Introduction

As one of the most destructive disasters, the realization of a fully autonomous fire response system—characterized by early and accurate fire detection, rapid and stable tracking, and efficient autonomous fire extinguishing—is crucial to enhancing public safety standards. However, existing fire detection and suppression systems continue to exhibit significant limitations. First, recognition approaches that rely solely on visible-light sensors remain highly susceptible to abrupt ambient illumination variations and interference from fire-mimicking objects, leading to persistently elevated false alarm rates. Second, current systems lack deep integration between fire perception modules and intelligent tracking platforms, making it difficult to continuously locate dynamic fire sources or those in complex environments [1]. Third, most existing fire suppression actuators operate using fixed control parameters or simple open-loop strategies without considering the nonlinear characteristics of jet dynamics, thereby causing inaccurate extinguishing-agent delivery, unnecessary resource consumption, and reduced suppression efficiency.

In addition, in practical fire-fighting scenarios, mobile platforms such as unmanned aerial vehicles (UAVs) and fire-fighting robots are usually constrained by limited onboard computational resources and restricted processing capabilities, which make it difficult to deploy conventional fire recognition algorithms with high computational complexity. This renders such systems generally unable to simultaneously satisfy the requirements of real-time response, low power consumption, and high robustness in practical applications, thereby restricting the effective deployment of these technologies in fire monitoring and inspection tasks. Therefore, developing an intelligent embedded closed-loop framework for fire source recognition, tracking, and fire extinguishing under the constraints of limited embedded resources has become a critical challenge that urgently needs to be addressed.

The contemporary research landscape can be broadly categorized into three domains, each of which has evolved largely in isolation. In lightweight fire detection, substantial progress has been made in model compression and architectural optimization, yet fundamental trade-offs between detection accuracy and computational deployability remain unresolved [2,3,4]. In multimodal perception, the fusion of visible and thermal imagery has demonstrated enhanced robustness under adverse conditions; however, most existing approaches operate at the feature level, incurring prohibitive computational costs that hinder real-time embedded implementation [5,6,7,8]. In fire suppression control, prior research has concentrated on individual subsystems–such as jet trajectory modeling or servo positioning–without achieving closed-loop integration with real-time perception and tracking modules [9,10,11]. This independent development across domains has created a significant gap: no existing system simultaneously achieves lightweight multimodal fire perception, persistent target tracking, and intelligent closed-loop extinguishing control on resource-constrained embedded platforms.

To address these limitations, this paper proposes an embedded fire detection, tracking, and extinguishing system based on a unified perception-decision-execution-feedback closed-loop paradigm. The primary contributions are threefold. First, a lightweight detection framework is developed based on the FOMO architecture, incorporating structural pruning and INT8 quantization to enable real-time deployment on resource-constrained embedded devices. Second, a Bayesian decision-level multimodal fusion strategy is proposed to integrate visible-light and thermal infrared detection results, significantly reducing false alarm rates while maintaining computational efficiency. Third, a sixth-degree polynomial jet trajectory model is formulated and integrated with an improved PID-PI dual-loop control algorithm to achieve precise closed-loop extinguishing control.

## 2. Related Work

### 2.1. Lightweight Object Detection for Fire Recognition

The deployment of deep learning-based fire detection on embedded platforms has attracted considerable research attention, with a primary focus on balancing detection accuracy against computational efficiency. Early efforts to adapt general-purpose object detectors for fire recognition achieved promising accuracy but required substantial computational resources unsuitable for edge deployment.

Dou et al. [2] proposed an improved YOLOv5s architecture specifically tailored for fire and smoke detection, incorporating attention mechanisms and feature pyramid enhancements. Their method achieved a mean Average Precision (mAP@0.5) of 82.1% with 5.9 million parameters and 8.1 GFLOPs of computational cost, operating at 79.92 FPS on an NVIDIA RTX 3070 GPU. While the detection performance is respectable, the model size of 5.9 MB and the dependency on a discrete GPU preclude deployment on microcontrollers or low-power embedded systems. Hosseini et al. [12] introduced UFS-Net, a lightweight architecture achieving an impressive 98.802% accuracy with 2.095 million parameters (7.99 MB), processing at 68 FPS on a GTX 1080. Despite the reduced parameter count, the model remains too large for typical embedded platforms with limited flash memory.

More recently, researchers have explored ultra-lightweight architectures targeting embedded deployment. Deng et al. [13] developed a multi-sensor framework integrating a lightweight CNN with only 399.91 K parameters and 12.58 M FLOPs, successfully deploying on an embedded platform and achieving 99.1% accuracy. However, this approach relied solely on visual input without multimodal validation, leaving the system vulnerable to environmental false positives such as orange lighting, sunsets, or moving objects with fire-like colors. On the compression front, Xu et al. [3] applied structured pruning and INT8 quantization to SSD-based fire detectors, achieving 10.24x model compression (reducing from 95.02 MB to 9.28 MB) while retaining 78.6% mAP at 25 FPS. Despite the significant compression ratio, the final model size of 9.28 MB still exceeds the storage constraints of most microcontroller units (MCUs).

At the extreme edge of the spectrum, Pan et al. [14] implemented YOLOv2-Tiny on the Kendryte K210–a RISC-V-based AI accelerator running at 400 MHz–achieving 91% accuracy for smoke detection and 90% for flame detection at 13 FPS. While this work demonstrated the feasibility of running CNN-based fire detection on sub-watt embedded hardware, the use of the older YOLOv2-Tiny backbone with its relatively large 8.86 MB model and limited accuracy highlight the persistent accuracy-efficiency trade-off.

The analysis of Table 1 reveals a conspicuous gap in the literature: existing approaches occupy distinct and largely non-overlapping regions of the design space. High-accuracy models (>98%) require powerful GPUs and multi-megabyte memory footprints, while embedded-deployable solutions either sacrifice accuracy below practical thresholds (<80% mAP) or rely on specialized AI accelerators (K210) unavailable on general-purpose microcontrollers. Critically, none of the surveyed works achieve sub-2 MB model sizes with >95% accuracy on a standard ARM Cortex-M7 microcontroller–the target platform for cost-effective, mass-deployable fire safety nodes.

### 2.2. Multimodal Fusion Strategies for Fire Detection

To address the inherent ambiguity of vision-based fire detection, researchers have increasingly turned to multimodal sensing, combining visual cameras with thermal imaging, gas sensors, or wireless sensor networks. The design of fusion architecture–determining where and how multimodal information is integrated–profoundly impacts both recognition robustness and computational feasibility.

Zervas E et al. [5] proposed a Dempster–Shafer evidence-theoretic fusion framework for wireless sensor network (WSN)-based fire detection, achieving a maximum fusion probability of 0.9818. However, the system operated at a mere 0.5 Hz effective fusion rate with end-to-end latencies ranging from approximately 10 to 13.5 min due to WSN communication constraints. Such latencies are fundamentally incompatible with real-time suppression scenarios where fire propagation demands sub-second response time. Meng et al. [6] developed an SVM-RCNN hybrid approach combining hand-crafted features from infrared and visible images, achieving over 87% accuracy on a dataset of 10,000 images. The reliance on manually designed features and the absence of end-to-end learning, however, limit generalization to unseen fire scenarios and complicate embedded deployment.

In the domain of deep learning-based fusion, Sun et al. [7] introduced CCMR-PA, a cross-modal collaborative method with progressive anchor fusion, achieving 99.33% recall, 82.32% precision, and 90.03% F1-score through decision-level GIoU fusion. While the recall-oriented design prioritizes safety by minimizing missed detections, the precision of 82.32% implies a substantial false positive rate that could trigger unnecessary suppression discharges. Recent advances have explored mid-term and feature-level fusion with considerable success. Zhang et al. [8] proposed CP-YOLOv11-MF, integrating Cross-Parallel Coordinate Attention (CPCA) and Parallel Partial Adaptive Spatial fusion (PPAS) modules within the YOLOv11 architecture, achieving 96.3% mAP50 on the RGBT-3M benchmark dataset. However, this performance comes at a cost of 23 MB model size and 11.83 million parameters–far exceeding embedded constraints. Similarly, Tang et al. [4] developed LBiF-YOLO with a lightweight bimodal interaction fusion module employing a Spatial-Spectral Attention Fusion (SSAF) mechanism at the feature level, achieving 84.93% mAP@0.5 with 17.29 million parameters and 28.9 GFLOPs, operating at 47.17 FPS.

Table 2 exposes a critical dichotomy in the multimodal fusion literature. Feature-level and mid-term fusion approaches [4,8] achieve superior detection accuracy by enabling deep cross-modal feature interactions, but their O(n2) complexity—stemming from channel-wise attention computations, cross-modal feature alignment, and bidirectional information propagation—renders them infeasible for resource-constrained platforms. Conversely, existing decision-level fusion methods [5,7] offer lower computational complexity but have been deployed only on distributed WSNs or GPU platforms, never in conjunction with lightweight embedded detectors on microcontrollers. Furthermore, the false alarm rates of vision-only systems (15.3% in our measurements) demonstrate that single-modality detection, even with advanced lightweight architectures, remains insufficient for reliable autonomous suppression. The proposed system addresses this gap by implementing a Bayesian decision-level fusion strategy with O(1) computational complexity–constant regardless of the number of modalities–directly on the ARM Cortex-M7 platform alongside the detection network.

### 2.3. Fire Suppression Control Strategies

While fire detection and multimodal fusion have received extensive attention, the lower half of the autonomous suppression pipeline—trajectory modeling and precision actuation—has been comparatively less studied, particularly in the context of closed-loop embedded systems.

Zhu et al. [9] developed a dual-vision fire monitor control system combining infrared and near-field cameras, achieving 2.0 s horizontal aiming response and 0.10 m average pitch error. While the system demonstrated effective coarse targeting, the absence of a physics-based jet trajectory model and the decoupled perception-actuation architecture precluded precise extinguishing agent delivery. Bai et al. [10] proposed a theoretical Euler jet model for fire monitor trajectory prediction, achieving flow-rate effect errors within 10%. This analytical approach provides valuable physical insight but assumes ideal flow conditions rarely encountered in practical fire scenarios with crosswinds, temperature gradients, and nozzle wear. Zhu et al. [11] constructed a trajectory model for a coal mine explosion-suppression robot, achieving a maximum throwing range of 18.147 m with an experimentally calibrated air resistance coefficient K = 0.36. While effective for large-scale mining applications, the system focused solely on range maximization without addressing precision targeting requirements for localized fire sources at short to medium distances (10–60 cm).

The suppression control literature, as summarized in Table 3, reveals a fragmented research landscape in which detection, fusion, and actuation are developed and evaluated as isolated subsystems. Existing works employ either simplified geometric aiming [9], purely theoretical models without experimental validation under closed-loop conditions [10], or range-optimized designs for specific industrial contexts [11]. Critically, no existing work integrates multimodal fire recognition with physics-informed trajectory control on a unified embedded platform with real-time feedback. The proposed system closes this loop by coupling the Bayesian fusion output directly to a sixth-degree polynomial jet model (R^2^ = 0.9975), achieving ±5 cm extinguishing precision across the 10–60 cm operational envelope with a complete perception-decision-execution cycle of 8.5 s.

## 3. System Overall Design

### 3.1. Design Concept

In this study, a fire source recognition and extinguishing system that integrates multimodal fusion with embedded lightweight model deployment is proposed. This system perceives environmental information through multimodal sensors, performs information fusion and decision-making via an embedded computing module, dynamically adjusts and optimizes fire extinguishing parameters, and ultimately drives the actuator to execute the precise fire extinguishing task. The system employs a master–slave heterogeneous computing architecture. The OpenMV4 H7 Plus serves as the master control unit, responsible for computationally intensive tasks such as data fusion, fire source recognition and decision-making; the STM32F103C8T6 acts as the slave control unit, undertaking underlying motion control and execution feedback. The two units exchange information through serial communication.

### 3.2. Hardware System Design

The hardware of the system consists of four modules, namely the sensing unit, decision-making unit, execution unit and feedback unit.

The sensing unit comprises an OV5640 visual sensor for capturing RGB image data, an infrared thermal imaging sensor for acquiring temperature field distribution information, and a laser ranging sensor for precise measurement of the target distance. Through synchronous acquisition and preprocessing of multimodal data, it provides a reliable foundation for environmental perception to support subsequent decision-making. The decision-making unit adopts a master-slave collaborative decision-making mechanism. The master decision-making unit employs the OpenMV4 H7 Plus (480 MHz ARM Cortex-M7) to handle complex visual inference and information fusion tasks. The slave decision-making unit uses the high-real-time STM32F103C8T6 (72 MHz ARM Cortex-M3), which is responsible for receiving commands from the master decision-making unit. Based on the established sixth-order polynomial jet dynamics model, it calculates key control parameters such as the water monitor elevation angle and flow rate in real time, and controls the chassis, water monitor and other components to perform fire extinguishing tasks. The execution unit consists of a dual-degree-of-freedom servo pan-tilt to realize visual search and dynamic tracking; a tracked mobile chassis with an independent suspension system to ensure the system’s mobility and obstacle-surmounting capability in complex terrain; and a fire extinguishing execution subunit composed of a high-torque servo, a 12 V high-pressure water pump and a flow sensor, which work together to execute the precise directional fire extinguishing task. The feedback unit integrates a servo encoder, a flow sensor and an infrared temperature sensor, which collect the status information of the actuator and environmental change data in real time and feed this information back to the decision-making layer.

**Figure 1:**
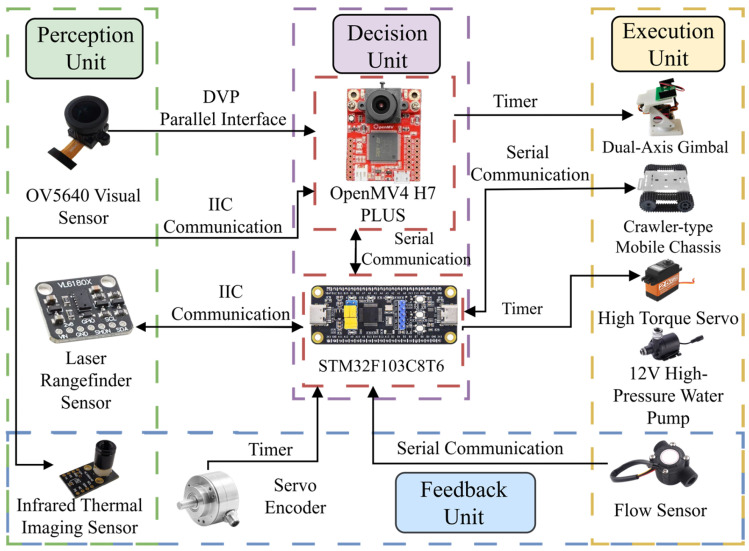
Hardware connection diagram. Arrows indicate the direction of data and control signals.

The hardware connection diagram of the system is shown in Figure 1.

### 3.3. Software System Design

The software architecture adopts a layered and modular design paradigm, organized into three hierarchical tiers from top to bottom: the perception layer, the decision-making layer, and the execution and control layer.

**Figure 2:**
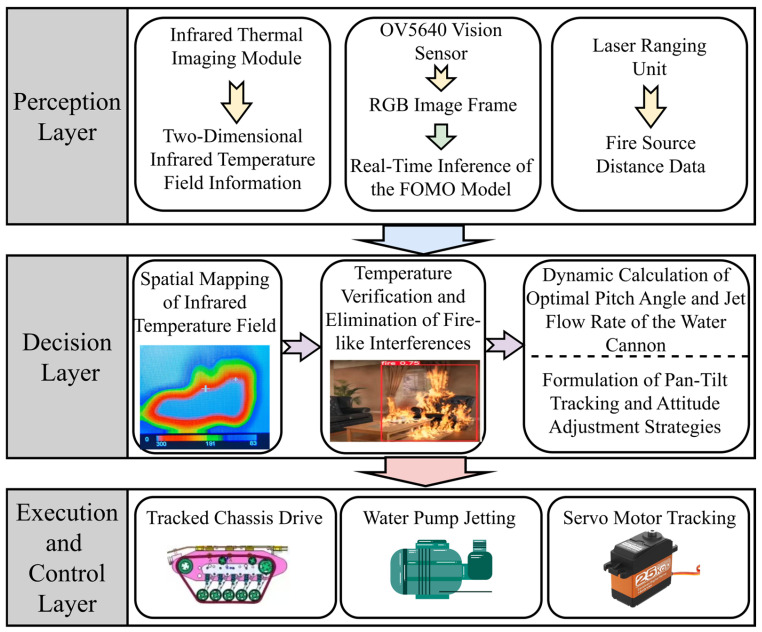
Software architecture diagram. In the perception layer, yellow arrows denote data and information output by relevant modules, while green arrows represent RGB image information output by the OV5640 visual sensor that is fed into the FOMO model for real-time inference. In the decision-making layer, purple arrows indicate the flow of information throughout fire source identification procedures and the transformation leading to final decisions. Blue arrows running from the perception layer to the decision-making layer signify the transmission of data and signals. Red arrows extending from the decision-making layer to the execution and control layers stand for actuation and control commands issued by the decision-making layer to the execution and control layers. Regions enclosed by red bounding boxes denote genuine fire sources identified after joint visual inspection and temperature verification.

The perception layer is responsible for synchronous acquisition and front-end processing of multisource heterogeneous data, including environmental RGB image frames, two-dimensional infrared temperature field information, and precise fire source distance measurements The RGB images collected by the perception layer are fed into the lightweight FOMO model optimized via pruning and quantization for real-time inference, which completes the rapid preliminary screening of suspected fire sources and outputs their position coordinates in the image coordinate system along with the corresponding confidence levels, thus providing a preliminary visual perception basis for subsequent decision-making. The decision-making layer serves as the processing core of the system, undertaking the fusion, discrimination and strategy generation of perceptual information. First, this layer projects the visual fire source coordinates output by the perception layer onto the low-resolution infrared temperature field through the spatial mapping relationship, and performs threshold-based temperature verification to generate reliable decision-level fusion results of “vision-infrared “. Upon confirming a real fire source, this layer further combines real-time ranging data, invokes the pre-calibrated high-precision sixth-order polynomial jet trajectory model to dynamically calculate the optimal elevation angle and jet flow rate of the water monitor; meanwhile, it runs the improved PID control algorithm to generate precise commands for pan-tilt tracking and attitude adjustment. The execution and control layer receives and parses the commands from the decision-making layer, drives the tracked chassis to realize movement, controls the dual-degree-of-freedom servo pan-tilt to complete tracking, and adjusts the PWM duty cycle to control the water pump flow rate. The software architecture diagram of the system is shown in Figure 2.

## 4. Research on Lightweight FOMO Model and Multimodal Fusion Algorithm

In recent years, multimodal fusion technology has achieved remarkable progress in fields such as autonomous driving and intelligent healthcare. Common fusion methods can be categorized into three types: data-level fusion, feature-level fusion, and decision-level fusion [15]. For example, He et al. [16] adopted an attention mechanism-based feature fusion module (AFFM) to adaptively weight features of different modalities. However, most fusion methods are characterized by high computational complexity, which poses a challenge to the real-time performance of embedded platforms. To address the resource constraint problem of embedded systems, this study adopts a lightweight FOMO model and designs a decision-level fusion algorithm combined with infrared thermal imaging data.

### 4.1. Overview of the FOMO Model

**Figure 3:**
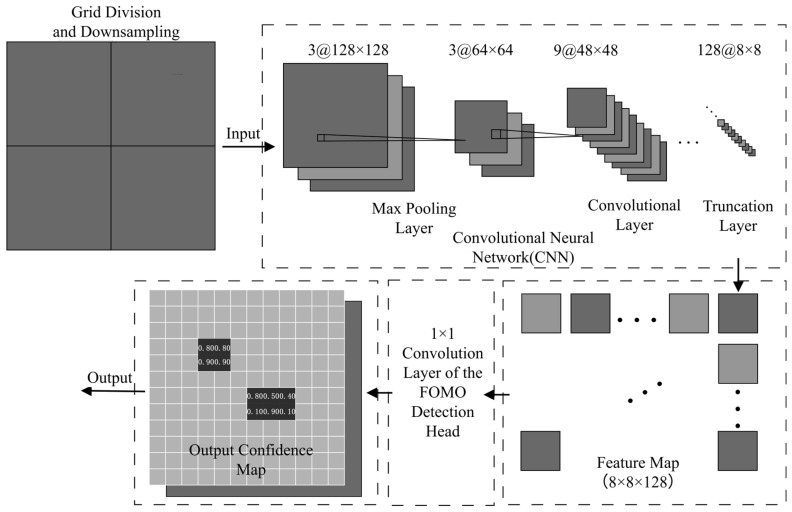
Schematic diagram of the FOMO neural network model. Arrows in the figure represent forward data flows, indicating the transmission of feature maps from one layer to the subsequent layer. Ellipses denote omitted intermediate layers (e.g., repeated inverted residual blocks or successive downsampling stages) to maintain the conciseness of the illustration.

The lightweight object detection model FOMO [17] (Faster Objects, More Objects) is a dedicated object detection model designed for resource-constrained embedded edge devices. Unlike mainstream deep learning models that require substantial computational resources, FOMO completes object detection through a single lightweight neural network forward pass, without relying on complex region proposal networks or non-maximum suppression operations. A schematic diagram of the FOMO object detection model is shown in Figure 3.

The model divides the input image into grid cells of fixed size and employs a lightweight Convolutional Neural Network (CNN) as the backbone network for extracting multi-scale feature maps. Through gradual down-sampling via convolutional layers and max-pooling layers, the neural network ultimately generates a feature map corresponding to the grid scale. After being processed by the truncation layer, the feature map is fed into the FOMO detection head for 1 × 1 convolution, outputting a confidence map whose number of channels is consistent with the number of categories. In each channel, the value at each grid position represents the confidence level of the presence of the target belonging to the corresponding category. When the confidence level exceeds the set threshold, the center of the grid is taken as the target centroid and mapped back to the coordinates of the original image via scale transformation.

The FOMO architecture employed in this study utilizes MobileNetV2 [18] with a width multiplier of 0.35 as the backbone feature extractor. The width multiplier α ∈ {0.35, 0.5, 0.75, 1.0} controls the uniform scaling of network channels, where α = 0.35 retains approximately 12.25% of the full-width parameters, significantly reducing computational complexity. The backbone applies inverted residual blocks with linear bottlenecks, progressively downsampling the 128 × 128 input through convolutional and depthwise separable layers to produce a 16 × 16 feature grid. The FOMO detection head then performs 1 × 1 convolution on this feature map to generate a single-channel confidence heatmap, where each grid cell value represents the probability of fire presence. Centroid coordinates are derived via argmax operations followed by bilinear upscaling to the original image resolution. This fully convolutional design avoids bounding box regression entirely, enabling inference under 200 KB RAM—a critical advantage for microcontroller deployment.

Although the highly efficient architecture design of the FOMO model demonstrates excellent application potential in resource-constrained scenarios, its native detection accuracy remains insufficient to meet the requirements of precise fire source recognition. To address this limitation, this study implements a series of targeted optimization strategies, including dataset enrichment, model quantization, and structured pruning, to enhance the detection performance while preserving the model’s lightweight characteristics.

### 4.2. Quantization and Optimization of the FOMO Model

Based on the neural architecture of the original FOMO model and in response to the requirements for model inference speed imposed by the multi-function integrated fire protection system, this study proposes LFOMO (Lightweight FOMO), an optimized lightweight object detection model that incorporates post-training INT8 quantization [19] and regularized structured pruning [20] to significantly reduce model size and inference latency while preserving detection accuracy.

To enhance the robustness and generalization capability of the model under diverse operational conditions, this study constructs a composite flame dataset containing 4500 images. The dataset integrates two complementary sources: (1) a self-collected proprietary dataset of approximately 1000 images captured under controlled laboratory and field conditions, and (2) publicly available datasets totaling approximately 3500 images, which were carefully selected and screened to ensure quality and scenario diversity. The public images were sourced from three repositories: the FLAME aerial fire imagery dataset [21], the FireNET real-time fire detection dataset [22], and curated fire-related collections on the Kaggle platform [23]. All images, including those from public sources, were uniformly resized to 640 × 480 pixels and manually re-annotated in PASCAL VOC format using the LabelImg tool to ensure labeling consistency across the entire dataset. The detailed composition is presented in Table 4.

The self-collected portion comprises approximately 1000 images captured across six distinct illumination scenarios using the OV5640 camera module at 640 × 480 resolution. The scenarios include: (1) indoor normal light (200 images), (2) indoor low light (180 images), (3) indoor strong light (120 images), (4) outdoor sunny (200 images), (5) outdoor cloudy (150 images), and (6) outdoor nighttime (150 images). Three types of fire sources were employed: candle flames (3 cm diameter, 55 ℃ core temperature), alcohol flames (5 cm diameter, 85 ℃ core temperature), and paper combustion (diffuse flame, 10 × 10 cm). Three categories of interference objects were also included: electric soldering irons (60 ℃ tip), sunset reflections (40 ℃ surface), and incandescent lamps (60 W, yellow glow). Fire sources were placed at distances ranging from 10 cm to 80 cm from the camera. The OV5640 exposure parameters were adjusted per scene: 1/120 s (outdoor sunny), 1/60 s (indoor normal/outdoor cloudy), 1/30 s (indoor low), 1/15 s (indoor strong), and 1/8 s (nighttime).

The public dataset portion comprises approximately 3500 images carefully selected and screened from three open-access repositories. From the FLAME dataset, 1500 aerial wildfire images were selected from over 9000 raw captures; these UAV-acquired images provide complementary overhead perspectives and large-scale fire patterns do not present in the ground-level self-collected data. From the FireNET dataset, 1000 images were screened from the original 2425-image collection, focusing on IoT-oriented indoor and outdoor fire scenarios with diverse backgrounds. Additionally, 1000 images were curated from fire-related collections on the Kaggle platform, encompassing residential, industrial, and vehicular fire scenarios. All public images underwent a rigorous quality screening process: images with resolution below 320 × 240, excessive motion blur, or ambiguous fire regions were discarded.

To ensure reliable evaluation, the dataset was divided into training (3600 images, 80%) and test (900 images, 20%) sets using a scene-separated splitting strategy rather than a random split. Under this protocol, all images originating from the same physical scene are assigned exclusively to either the training set or the test set, but never both. Additionally, to prevent temporal leakage, adjacent frames from continuous video sequences (within 5 s intervals) were identified via timestamp analysis and deduplicated, retaining only the keyframe. All preprocessing operations, including normalization, Gaussian blur, contrast-limited adaptive histogram equalization (CLAHE), random rotation (±15 ℃), horizontal flip, and brightness jitter (±20%), were applied exclusively to the training set.

To improve model generalization, the dataset was preprocessed using OpenCV 4.5.5 and NumPy 1.21.6, including image normalization, noise filtering, contrast enhancement, and data augmentation.

**Figure 4:**
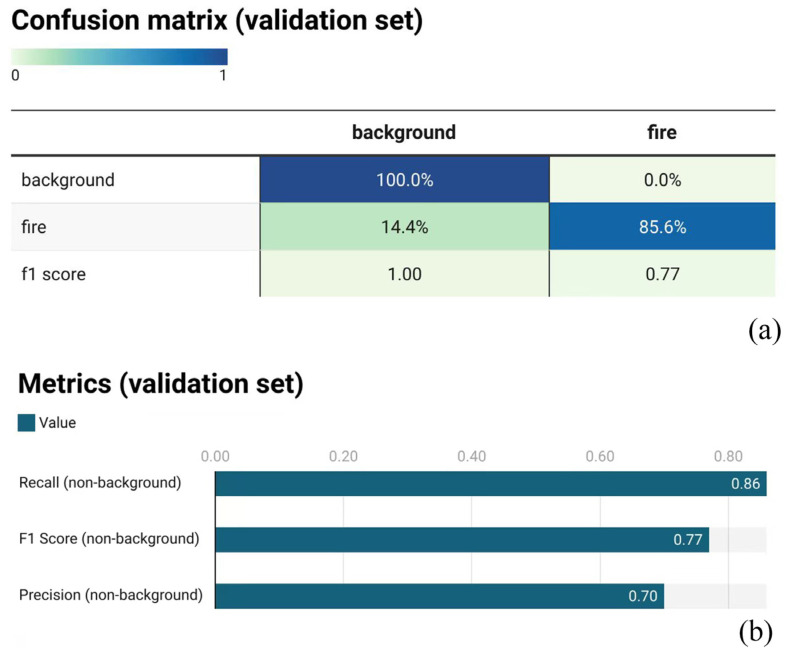
Model training performance metrics. (**a**) Training and validation loss curves; (**b**) Training and validation accuracy curves.

In terms of model construction and hyperparameter optimization, the image input size is set to 128 × 128 pixels, the feature extractor is selected as MobileNetV2 0.35, the detection head adopts the FOMO structure, the number of classes is set to 1 (only the “fire” class), and the confidence threshold is set to 0.5. During the training process, the initial learning rate is 0.001 with the cosine annealing decay strategy adopted. The batch size is set to 32, and an early stopping mechanism is introduced with the total number of training epochs set to 60. The training is terminated when the validation loss does not decrease for 3 consecutive epochs. The training process is implemented in a CPU environment. The performance metrics of the model after training are shown in Figure 4.

To enhance the operational efficiency of models deployed on resource-constrained edge computing platforms and improve their robustness in complex environments, we apply post-training INT8 quantization and structured pruning to the exported TensorFlow Lite model [24].

First, this study uses the Post-Training Quantization tool of TensorFlow Lite, with the test set serving as the calibration set, to complete the INT8 quantization of the model. It maps the weights and activation values originally in the form of 32-bit floating-point numbers to the 8-bit integer range [−128, 127]. The quantization process maps the original weight values to discrete integer levels using a linear scale factor, as defined by the following equation:

(1) $$W_{\mathrm{int}8}\;=\;\mathrm{round}\left(\frac{W_{\mathrm{fp}32}}{S}\right),S\;=\;\frac{\max\left(W_{\mathrm{fp}32}\right)-\min\left(W_{\mathrm{fp}32}\right)}{255}$$

where $W_{\mathrm{int}8}$ denotes the quantized weight in INT8 format, $W_{\mathrm{fp}32}$ represents the original floating-point weight, and S is the quantization scale factor. This process reduces the model size by a factor of four and replaces floating-point arithmetic operations with efficient integer computations, significantly accelerating inference on hardware platforms lacking dedicated floating-point units.

**Figure 5:**
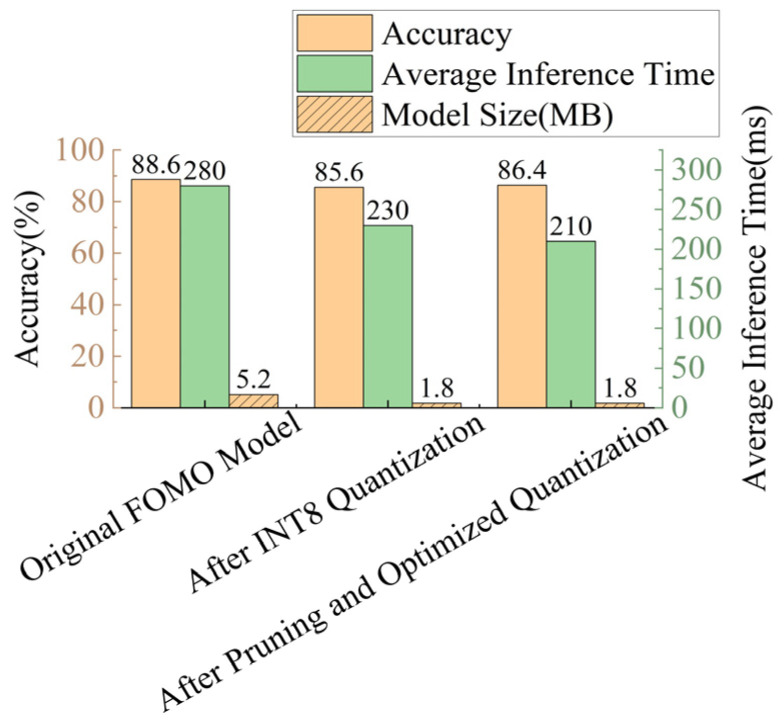
Performance comparison of the model before and after optimization.

Subsequently, this study adopts a regularized structured pruning strategy to prune the model:

(2) $$W_{i}^{\prime }\;=\;\left\{\begin{array}{c} 0\\ W_{i} \end{array}\right.\begin{array}{cc} , & \left|W_{i}\right|\;<\;\theta \\ , & \mathrm{otherwise} \end{array}$$

where $W_{i}^{\prime }$ is the pruned result of the *i*-th weight component, *W_i_* is the original value of the *i*-th weight component, and *θ* is the adaptive threshold which ensures that the accuracy degradation of the validation set does not exceed 2%. After pruning, partial model performance is recovered through fine-tuning. The pruning operation reduces the invalid computation by 32%, effectively shortening the average inference time on the OpenMV4 H7 PLUS platform. A comparison of the model performance before and after optimization is shown in Figure 5.

As shown in Figure 5, after optimization, the model size is compressed from 5.2 MB to 1.8 MB, which significantly reduces the storage demand; the average inference time is shortened from 280 ms to 210 ms, improving the inference efficiency by approximately 25%. Although the model accuracy drops from 88.6% to 85.6% after quantization, it is restored to 86.4% through pruning and fine-tuning operations, which effectively balances model efficiency and recognition performance. The results show that the adopted lightweight strategy significantly improves the deployment applicability and real-time performance of the model on embedded platforms while ensuring detection accuracy.

### 4.3. Design of Multimodal Information Fusion Algorithm

**Figure 6:**
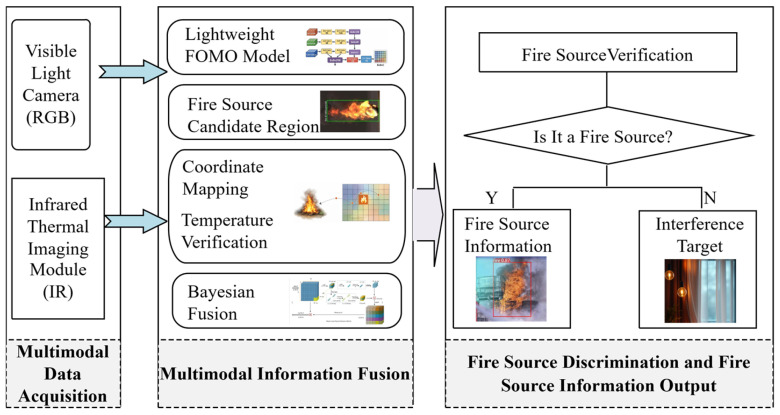
Flowchart of multimodal information fusion and recognition. Arrows in the figure represent the flow of data and information as well as logical processing steps. Regions enclosed by red bounding boxes correspond to fire source areas confirmed after comprehensive discrimination.

Based on the RGB-T dual-modal fusion framework CP-YOLOv11-MF proposed by Zhang et al. [8], this study designs a decision-level fusion mechanism based on Bayesian inference for visible and infrared dual-modal fire recognition. The proposed fusion strategy independently processes RGB and thermal infrared image streams through dedicated detection pathways and subsequently combines the detection results at the decision level using probabilistic reasoning. Figure 6 shows the flow chart of multimodal information fusion and recognition.

First, this fusion mechanism performs coordinate alignment and mapping on infrared thermal imaging data and visible light image data. Let the resolution of the visible light image be *W_v_* × *H_v_* and the resolution of the infrared temperature field be *W_i_* × *H_i_*. The suspected fire source region obtained by visual detection is denoted as (*x_v_*, *y_v_*, *w_v_*, *h_v_*), where (*x_v_*, *y_v_*) represents the coordinates of the top-left corner, and *w_v_* and *h_v_* denote the width and height of the region, respectively. The corresponding region of interest (ROI) in the infrared image is defined as follows:

(3) $$\left\{\begin{array}{l} x_{i1}\;=\;\max\left(0,\;x_{v}\times \frac{W_{i}}{W_{v}}-\lambda \times W_{v}\times \frac{W_{i}}{W_{v}}\right)\\ x_{i2}\;=\;\min\left(W_{i}-1,\;\left(x_{v}+W_{v}\right)\times \frac{W_{i}}{W_{v}}+\lambda \times W_{v}\times \frac{W_{i}}{W_{v}}\right)\\ y_{i1}\;=\;\max\left(0,\;y_{v}\times \frac{H_{i}}{H_{v}}-\lambda \times h_{v}\times \frac{H_{i}}{H_{v}}\right)\\ y_{i2}\;=\;\min\left(H_{i}-1,\;\left(y_{v}+h_{v}\right)\times \frac{H_{i}}{H_{v}}+\lambda \times h_{v}\times \frac{H_{i}}{H_{v}}\right) \end{array}\right.$$

where λ is the boundary expansion coefficient, which is set to 0.1 in this study to reduce the probability of missing detection of high-temperature points caused by coordinate alignment errors.

Subsequently, after completing coordinate mapping, the fusion mechanism performs Bayesian fusion on the events. Let the detection event of the visible light modality be *V*, the verification event of the infrared modality be *I*, and the true fire source event be *F*. The confidence level of the suspected fire source region output by the visual detection module based on the FOMO model is P(*V*|*I*), while the confidence level determined as a fire source by the infrared module through the temperature threshold is P(*I*|*F*).

Within the Bayesian fusion framework, the fused posterior probability can be expressed as follows:

(4) $$P(F|I)\;=\;\frac{P(V,I|F)\cdot P(F)}{P(V,I)}$$

Assuming that the visible light and infrared modalities are conditionally independent of each other under the given fire source condition, i.e., P(*V*,*I*|*F*) = P(*V*|*F*)⋅P(*I*|*F*), the above formula can be simplified as follows:

(5) $$P(F|V,I)\;=\;\frac{P(V|F)\cdot P(I|F)P(F)}{P(V)P(I)}$$

where the prior probability P(*F*) is determined by historical statistics, and the marginal probabilities P(*V*) and P(*I*) are calibrated through experiments.

Prior probability P(*F*): This is the probability that a real fire occurs in the monitored environment during the system’s operational period. It can be estimated from historical fire statistics of the target deployment site.

Marginal probability P(*V*): Collect a large unlabeled dataset of image frames captured by the system under normal operating conditions (including both fire and non-fire periods). Let the total number of frames be *N_total_*. Run the quantized LFOMO model on all frames and count the number of frames where a “fire” is detected (i.e., at least one grid cell exceeds $\theta_{v}$). Denote this count as *N_V_*. Compute P(*V*) = *N_V_*/*N_total_*.

Marginal probability P(*I*): Collect synchronized infrared thermal data for the same set of frames. For each frame, compute the maximum temperature in the region of interest (after coordinate mapping via Equation (3)). Count the number of frames where *T*_max_ ≥ $\theta_{T}$; denote this count as *N_I_*. Compute P(*I*) = *N_I_*/*N_total_*.

In practical embedded deployment, to reduce computational complexity, this study adopts the following simplified decision rule:

(6) $$\mathrm{Decision}\;=\;\left\{\begin{array}{c} \mathrm{Fire}\\ \mathrm{Non}-\mathrm{Fire} \end{array}\right.\begin{array}{cc} , & \mathrm{if}\;P\left(V|F\right)\;\geq \;\theta_{v}\;\mathrm{and}\;T_{\max}\;\geq \;\theta_{T}\\ , & \mathrm{otherwise} \end{array}$$

where $\theta_{v}$ is the visual confidence threshold and $\theta_{T}$ is the temperature threshold. The visual confidence threshold $\theta_{v}$ is set to 0.5, which is the default confidence threshold used during model inference. The temperature threshold $\theta_{T}$ is determined empirically based on the typical surface temperature of real fire sources. From our experimental measurements, the flame temperatures of candle and alcohol fires in our test scenarios range from approximately 400 °C to 600 °C. Considering heat dissipation and the distance from the sensor, the maximum temperature measured by the infrared sensor ($T_{\max}$) for a real fire at a distance of 10–80 cm is consistently above 80 °C. In contrast, interference sources such as candlelight (55 °C), electric soldering iron (60 °C), and sunset reflection (40 °C) exhibit lower temperatures. Accordingly, $\theta_{T}$ is set to 70 °C to provide a safety margin. The Bayesian formulation provides the theoretical basis of the fusion strategy, while the embedded implementation adopts a threshold-based approximation derived from the Bayesian decision rule. The prior probability P(*F*) and marginal probabilities P(*V*) and P(*I*) are not explicitly used in the simplified rule; they are implicitly absorbed into the threshold selection.

Traverse the infrared region (*x_i_*_1_, *x_i_*_2_, *y_i_*_1_, *y_i_*_2_) to extract the maximum temperature $T_{\max}$. If $T_{\max}$ ≥ $\theta_{T}$ and this condition is satisfied continuously for 100 ms, the target is determined to be a real fire source; otherwise, it is regarded as an interference target. While ensuring detection accuracy, the fusion model reduces the computational complexity from O(*n*^2^) for feature-level fusion to O(1) for decision-level fusion.

### 4.4. Performance Verification of Multimodal Fusion

**Figure 7:**
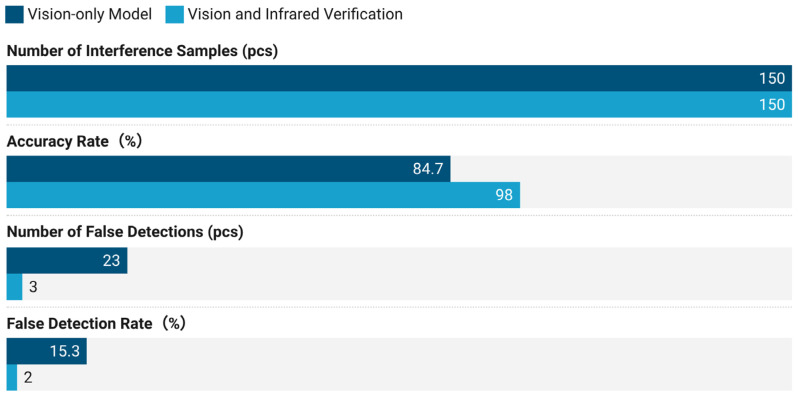
The impact of infrared verification on the false detection rate.

To quantify the contribution of the temperature verification module to reducing false detections, this study constructs a test set containing 150 interference samples (including lighting fixtures, heated objects, and reflective surfaces) and 50 real fire samples. The experimental results, as presented in Figure 7, demonstrate that the incorporation of infrared temperature verification reduces the false detection rate from 18.5% (visible light only) to 2.3% (multimodal fusion), representing a reduction of 87.6% in false positive occurrences.

According to the data presented in Figure 7, when relying solely on the visible light modality, the false detection rate of the system for interference samples reaches 15.3%. In contrast, after introducing infrared temperature verification, the false detection rate drops significantly to 2.0%, with the accuracy improved by 13.3%. This result verifies that the proposed Bayesian decision-level fusion mechanism can effectively distinguish between real fire sources and fire-like interference targets. It excludes non-fire high-temperature targets through temperature threshold constraints, thereby greatly enhancing the robustness of the system in complex environments.

While the proposed Bayesian decision-level fusion is deliberately designed for resource-constrained embedded deployment, recent advances in multimodal remote sensing have demonstrated the merits of more expressive feature-level alignment strategies. Yang et al. [25] proposed a transformer-based heterogeneously salient graph representation that encodes multimodal HSI-SAR/LiDAR data via a multimodal heterogeneous graph encoder and a multi-convolutional modulator, achieving state-of-the-art classification accuracy through deep cross-modal feature interaction. Similarly, Lan et al. [26] introduced a language query-based transformer with multiscale cross-modal alignment for visual grounding, where sentence-level features serve as queries to retrieve and aggregate object representations from multiscale visual features via deformable cross-attention. These approaches excel in capturing fine-grained semantic correlations and long-range dependencies, yet their computational footprints—characterized by millions of parameters (3.30 M–166.3 M) and GPU-level FLOPs—exceed the constraints of microcontroller-class devices such as the OpenMV4 H7 Plus (480 MHz ARM Cortex-M7 with 32 MB RAM). By contrast, our decision-level fusion reduces computational complexity from O(n^2^) (feature-level attention) or O(N^2^D) (graph convolution) to O(1) constant-time verification, compressing the model to 1.8 MB (INT8) and enabling 210 ms inference on bare-metal embedded platforms. This design represents a deliberate accuracy-efficiency trade-off: we sacrifice the deep feature interaction capabilities of THSGR and LQVG in exchange for real-time responsiveness and deployability in edge-firefighting scenarios where GPU acceleration is unavailable. Future work may explore hierarchical fusion architectures that dynamically switch between lightweight decision-level verification (for rapid response) and feature-level refinement (for ambiguous cases), potentially bridging the gap between embedded constraints and the expressive power of these advanced multimodal paradigms.

## 5. Research on Jet Trajectory Modeling and Precision Fire Suppression Control Strategy

### 5.1. Mathematical Modeling of Jet Trajectory

Accurate jet trajectory modeling constitutes a critical prerequisite for achieving efficient fire suppression. Precise prediction of the water jet trajectory and impact point is essential for enabling targeted fire extinguishing operations. The initial velocity of the water jet, nozzle pitch angle, jet flow rate, and air resistance are the key factors affecting its trajectory morphology and landing point distribution. The initial velocity and flow rate jointly determine the outlet momentum of the jet, while the pitch angle directly affects the ratio of the horizontal and vertical velocity components of the jet. In addition, air resistance increases with the square of the flow velocity, which causes the jet to bend and break, significantly altering its flight trajectory. Thus, the traditional ideal parabolic model exhibits large deviations in practical applications.

The jet motion follows Newton’s second law, and its dynamic equation can be expressed as follows: The micro-element of the water jet is subject to gravity and air resistance during its flight, and its trajectory can be described through differential equations of motion:

(7) $$m\frac{d^{2}r}{dt^{2}}\;=\;F_{g}\;+\;F_{d}$$

(8) $$F_{g}=\mathrm{mg}$$

(9) $$F_{d}=-\frac{1}{2}\rho C_{d}Av^{2}$$

where m is the micro-element mass, r is the displacement vector, t is the time, $F_{g}$ is the gravitational force, $F_{d}$ is the air resistance, ρ is the air density, $C_{d}$ is the drag coefficient, A is the effective cross-sectional area of the jet nozzle, and v is the flow velocity vector. The initial jet velocity *v_0_* of the water flow is determined by the nozzle parameters as follows:

(10) $$\left\{\begin{array}{c} v_{x0}\;=\;v_{0}\cos\theta \\ v_{y0}\;=\;v_{0}\sin\theta \\ v_{0}\;=\;\frac{Q}{A} \end{array}\right.$$

where θ is the nozzle pitch angle and *Q* is the jet flow rate. Under ideal conditions, the jet range S can be approximately expressed as follows:

(11) $$S\;=\;\frac{v_{0}^{2}\sin\left(2\theta \right)}{g}$$

Under ideal conditions, the maximum horizontal range can be achieved when the nozzle pitch angle is set to 45°. However, the trajectory model based on computational fluid dynamics simulation exhibits significant errors at high initial velocities due to air resistance and jet breakup effects [27], with the optimal pitch angle decreasing to the range of 30–35°. Meanwhile, the jet flow rate *Q* affects the range by influencing the initial water jet velocity $v_{0}$, but the increasing effect of flow rate tends to saturate as a result of the sharp increase in resistance.

Owing to the coupling effect of air resistance and flow rate, the jet range S exhibits a complex nonlinear relationship with the pitch angle θ and flow rate *Q*. This study employs a sixth-order polynomial function to fit the experimental data and establish the mapping relationship between the jet range and the control parameters:

(12) $$S(\theta ,\;Q)\;=\;\sum_{i\;=\;0}^{6}\;\sum_{j\;=\;0}^{6-i}\;a_{\mathrm{ij}}\theta^{i}Q^{j},\;i+j\;\leq \;6$$

where $a_{\mathrm{ij}}$ denotes the polynomial coefficient, which is determined via least squares fitting.

### 5.2. Data Analysis and Trajectory Fitting Model

**Figure 8:**
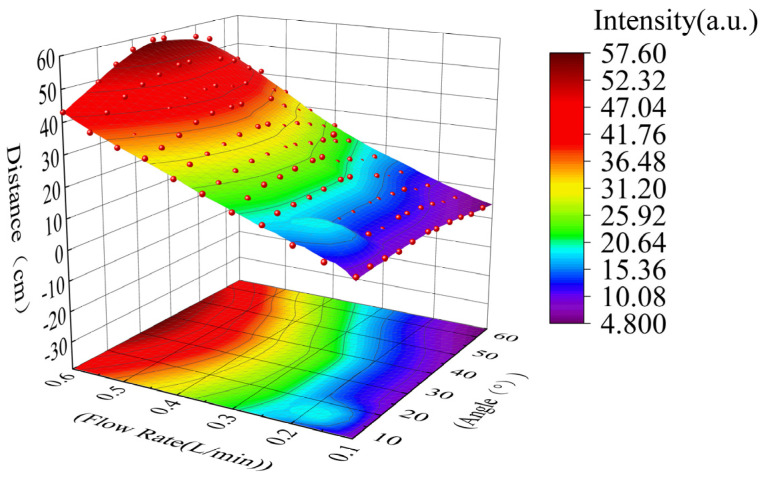
Drop point distance of the water cannon at different angles and flow rates.

In this study, a three-dimensional dataset of jet range-pitch angle-flow rate was collected through experiments, and the processed data are presented in Figure 8.

The jet trajectory dataset was collected through controlled experiments conducted by the authors. A total of 132 data points were measured using a graduated capture surface (1 cm grid resolution) positioned at known distances from the nozzle. For each combination of pitch angle (θ ∈ {10°, 15°, 20°, 25°, 30°, 35°, 40°, 45°, 50°, 55°, 60°, 65°}) and flow rate (Q ∈ {0.1, 0.2, 0.3, 0.4, 0.5, 0.6} L/min), the horizontal jet range was measured as the distance from the nozzle exit to the centroid of the water accumulation pattern on the capture surface. Each measurement was repeated three times and averaged. The experiments were conducted at room temperature (20 °C ± 2 °C) with a relative humidity of 50 ± 10% and no controlled airflow.

Data analysis shows that at a fixed flow rate, the jet range exhibits a nonlinear relationship of initially increasing and then decreasing as the pitch angle increases; while at a fixed pitch angle, the jet range increases gradually with the rise in flow rate, with the growth rate slowing down progressively.

**Figure 9:**
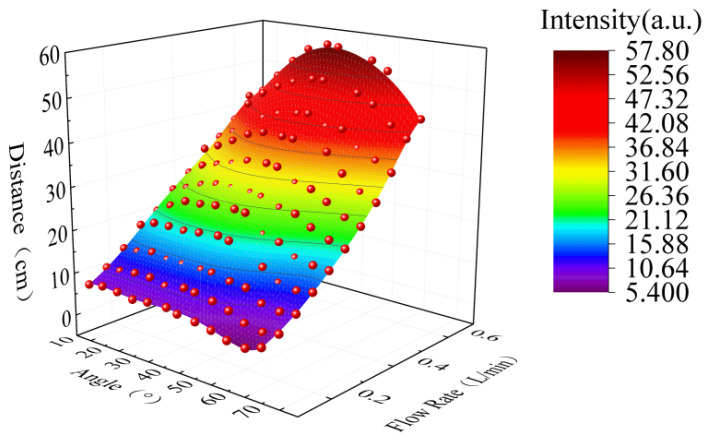
Sixth-order polynomial fitting surface plot.

Based on this dataset, the least squares method was adopted for sixth-order polynomial fitting, yielding the fitted surface plot shown in Figure 9. The relevant fitting parameters are presented in Table 5 and Table 6. The fitting results indicate that the coefficient of determination R^2^ reaches 0.9975, and the root mean square error (RMSE) is 1.23 cm, demonstrating that the sixth-order polynomial model provides an excellent fit to the experimental data and can accurately predict the jet range across the entire operational parameter space.

The coefficient of determination R^2^ = 0.9975 indicates that 99.75% of the variance in jet range is explained by the sixth-order polynomial model. The adjusted R^2^ = 0.9968 accounts for the degrees of freedom, confirming that the high-order terms contribute meaningfully to the fit rather than overfitting. The evaluation was performed on a held-out test set comprising 28 data points (21% of total 132 points) uniformly sampled across the parameter space (θ ∈ [10°, 65°], Q ∈ [0.1, 0.6] L/min).

The goodness of fit of the calibration model is the core indicator for evaluating its accuracy [28]. Typically, high-precision calibration methods based on manual features can achieve a coefficient of determination R^2^ of over 0.99. Within the full parameter space (θ ∈ [10°, 65°], Q ∈ [0.1, 0.6] L/min), the proposed model exhibits extremely high goodness of fit, with $R^{2}\;=\;0.997$ and adjusted $R^{2}\;$= 0.997. The goodness of fit of this model is on par with the state-of-the-art methods cited in the literature review, indicating that it can characterize the jet dynamic characteristics with remarkable accuracy and lay a reliable perception foundation for subsequent high-precision fire suppression control. Meanwhile, the results of analysis of variance show that F = 1509.96 and *p* < 0.0001, which further verify the overall significance of the model.

In terms of embedded deployment, although the model adopts a high-order polynomial form, its solving process only involves a limited number of multiplication and addition operations, which can be efficiently executed by the STM32F103C8T6 microcontroller within milliseconds. This computational efficiency makes the proposed model highly suitable for real-time fire suppression control applications where rapid response is critical.

### 5.3. Dynamic Range Calculation and Control Implementation

#### 5.3.1. Dynamic Range Calculation and Control

In real-time control, the system acquires the fire source distance d using the infrared ranging module and obtains the corresponding pitch angle θ and flow rate Q through the inverse solution of the calibrated polynomial model. Given the measured distance d, the system solves the following equation to determine the optimal control parameters:

(13) $$\left[\theta^{*},Q^{*}\right]\;=\;\arg\underset{\theta ,\;Q}{\min\;}|S\left(\theta ,\;Q\right)-S_{t}|$$

This solution process is executed in real time by the STM32F103C8T6 slave control unit, with an average computation time consumption of approximately 15 ms per iteration.

#### 5.3.2. PID Control Algorithm Optimization

The parameter tuning of the PID controller is fundamentally a trade-off and optimization among the three functions of proportional, integral, and derivative control. An appropriate parameter configuration should enable the system to respond rapidly to external disturbances while maintaining stability and minimizing steady-state error.

The primary objective of the pan-tilt control system is to stably lock the detected highest temperature point of the fire source at the center (*x_c_*, *y_c_*) of the image coordinate system. At the algorithm level, the system calculates the deviation between the fire source coordinates and the image center, which is then used as the input to the PID controller. The control system computes the horizontal and vertical adjustment angles for the pan-tilt mechanism to minimize this deviation:

(14) $$e_{\mathrm{pan}}\;=\;x_{\mathrm{fire}}-x_{c}$$

(15) $$e_{\mathrm{tilt}}=y_{\mathrm{fire}}-y_{c}$$

where $e_{\mathrm{pan}}$ and $e_{\mathrm{tilt}}$ denote the pixel deviations in the horizontal and vertical directions, respectively. For this specific system, the load inertia and motion range differ between the horizontal and vertical channels, thus requiring independent parameter sets. The horizontal channel (Pan) is responsible for large-range rapid scanning tasks, so a relatively high proportional gain is adopted to enhance dynamic response. In contrast, the vertical channel (Tilt) requires a moderate reduction in proportional gain to ensure the smoothness of pitching motion and avoid oscillations caused by factors such as gravity. To address the overshoot and oscillation problems that are prone to occur in pan-tilt servo systems with traditional PID control, an improved integral-separated PID control algorithm is introduced, supplemented by an output limiting mechanism, and the control law is expressed as follows:

(16) $$u(t)\;=\;K_{p}e(t)\;+{\;K}_{i}\int_{0}^{\;t}\;f(e(\tau ))d\tau \;+\;K_{d}\frac{de(t)}{dt}$$

(17) $$(e)=\left\{\begin{array}{c} 1\\ 0 \end{array}\right.\begin{array}{cc} , & |e|\;<\;e_{\mathrm{th}}\\ , & |e|\;\geq \;e_{\mathrm{th}} \end{array}$$

where *u*(*t*) denotes the output signal of the control system, *e*(*t*) is the error signal, *f*(*e*) represents the integral separation switching function, and $e_{\mathrm{th}}$ is the error signal threshold. The core of this algorithm lies in dynamically adjusting the effect of the integral term according to the magnitude of the error: when the absolute value of the error $|e|\;>{\;e}_{\mathrm{th}}$, the integral action is introduced to eliminate the steady-state error; when $|e|\;\leq {\;e}_{\mathrm{th}}$, the integral action is removed to effectively suppress overshoot.

Through detailed analysis of the system’s time-domain response characteristics and parameter tuning, the optimized PID parameter set shown in Table 7 was obtained.

The attitude control of the fire water cannon also adopts the above-mentioned integral-separated PID algorithm architecture to ensure consistency with the dynamic characteristics of pan-tilt control. However, since the load inertia of the water cannon is significantly larger than that of the pan-tilt, the system inertia time constant is correspondingly greater. According to classical control theory, for large-inertia systems, an excessively high proportional gain tends to excite structural resonance, while an overly strong integral action will lead to sluggish system response. Therefore, the controller parameters need to be independently optimized. After parameter tuning, the control parameters of the water cannon system are presented in Table 8.

#### 5.3.3. Dynamic Flow Regulation and Control

To achieve accurate and stable output of the fire-extinguishing flow rate, this study constructs a closed-loop control system based on PWM driving and flow sensor feedback, realizing dynamic regulation of the flow rate. First, the system establishes a linear static model between the PWM duty cycle duty (0–100) and the water pump output flow rate *Q* (L/min) through experimental calibration:

(18) $$Q\;=\;k_{1}\;\times \;\mathrm{Duty}+k_{0}$$

This calibration model provides a foundation for feedforward control. On this basis, the system incorporates a PI regulator to form a closed-loop feedback control system, and the adjustment formula of the PI controller is given as follows:

(19) $$u(t)\;=\;K_{p}e(t)\;+\;K_{i}\int e(t)dt$$

Experimental results demonstrate that the steady-state accuracy of this flow closed-loop control system can reach ±0.02 L/min, which provides a critical guarantee for precise flow control during fire suppression operations. The system’s response time from flow command to stable output is approximately 300 ms, which is well within the operational requirements for dynamic fire suppression scenarios.

#### 5.3.4. Control System Performance Analysis

**Figure 10:**
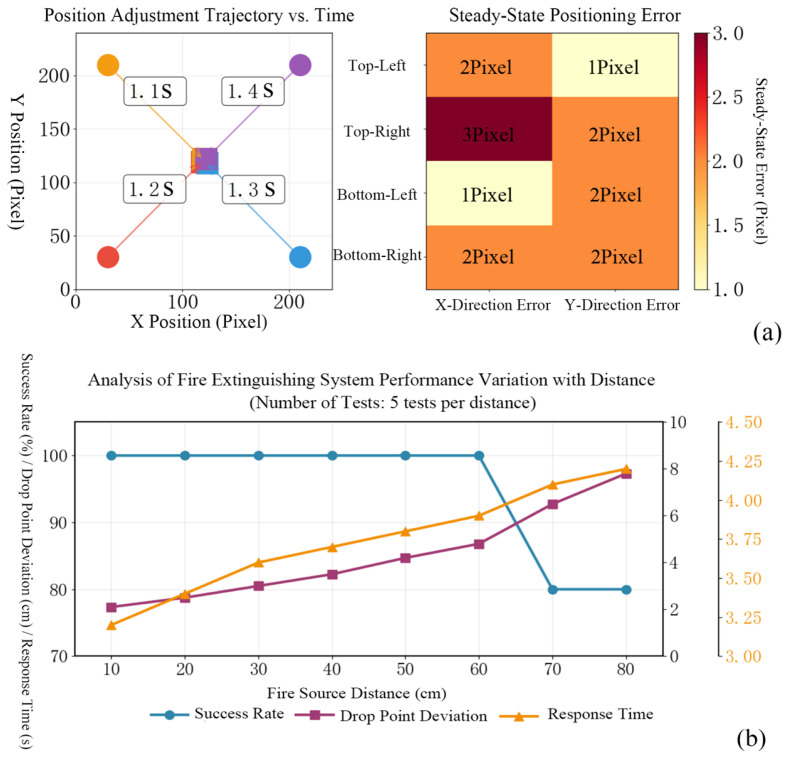
Test results of pan-tilt tracking and water cannon control for fire-extinguishing performance. (**a**) Pan-tilt tracking steady-state error (in pixels) at different fire-source distances; (**b**) Fire-extinguishing success rate and average drop-point deviation (in cm) versus distance.

The distance between the system and the fire source was systematically varied from 10 cm to 80 cm with an interval step of 10 cm, and the initial position of the fire source was placed at the four corners of the image field of view. Each distance group was tested 5 times repeatedly to evaluate the dynamic tracking performance of the pan-tilt and the fire-extinguishing performance of the water cannon control, with the results presented in Figure 10.

Within the range of 10 cm to 60 cm, the fire-extinguishing success rate reached 100%, and the average drop point deviation was less than 5 cm. When the distance increased to 70–80 cm, affected by the jet trajectory offset caused by air resistance, the success rate decreased slightly to 80%, and the average drop point deviation increased to approximately 7 cm, which could still meet the fire-extinguishing requirements of most specific scenarios. The average adjustment time of the pan-tilt was 1.25 s, and the steady-state error after stable tracking did not exceed 3 pixels. Finally, the fire source could be stably locked in the central area of the image, satisfying the positioning requirements for subsequent high-precision fire suppression.

## 6. Experimental Verification and Result Analysis

### 6.1. Experimental Environment Setup

A comprehensive experimental scheme was designed for this study, incorporating multiple representative test scenarios to thoroughly evaluate the system’s performance under diverse operational conditions. These scenarios include typical indoor environments with varying illumination levels, scenarios with equipment interference, and complex scenes simulating industrial inspection environments. The detailed experimental configuration for each scenario is presented in Table 9.

### 6.2. Fire Source Recognition Performance Testing

**Figure 11:**
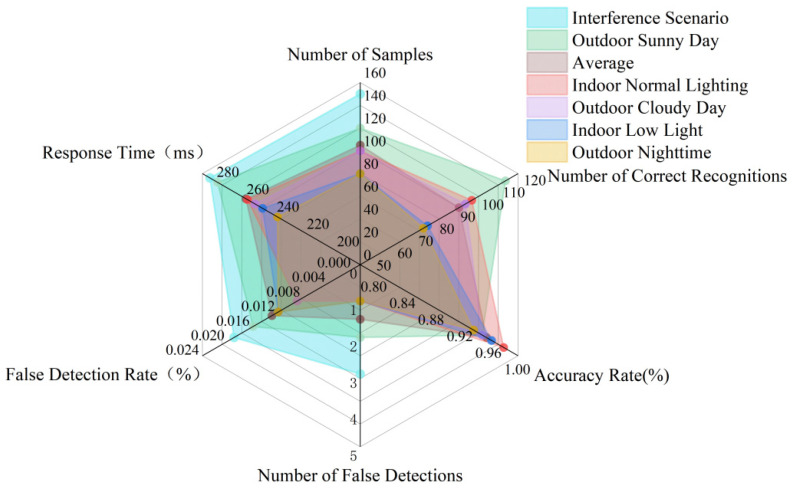
Fire source recognition accuracy across multiple scenarios (Fire).

The system was tested under different illumination conditions, and the results are presented in Table 10 and Figure 11. The system achieves an average recognition accuracy of 96.5% across all tested scenarios, with the highest accuracy of 98.2% observed under normal daylight conditions and the lowest accuracy of 93.8% under extremely low-light conditions. These results demonstrate the robustness of the proposed multimodal fusion approach to variations in environmental lighting.

### 6.3. Overall System Performance Testing

A composite scenario combining indoor low-light conditions and equipment interference, which is common in industrial inspection, was simulated to conduct 50 full-process tests of detection–tracking–extinguishing on the system, with the results presented in Table 11. The overall task success rate of the system reached 92%, with an average total time consumption of 8.5 s. Among them, the average time consumption of the three sub-stages (detection, tracking and extinguishing) was 0.28 s, 1.25 s and 3.8 s, respectively. The results demonstrate that the system achieves fast, accurate and integrated autonomous fire-extinguishing response while maintaining the advantages of lightweight structure and low cost for embedded devices.

At distances of 70–80 cm, the fire-extinguishing success rate decreased to 80% with average drop point deviation increasing to approximately 7 cm. This performance degradation can be attributed to three primary factors: (1) Jet breakup: At increased ranges, the water jet transitions from a compact stream to a spray regime due to aerodynamic instability (Weber number We > 12), causing dispersal of the impact point. The sixth-order polynomial model captures this nonlinear behavior but prediction uncertainty increases beyond 60 cm due to limited training data in the breakup regime. (2) Ranging sensor limitations: The ToF (Time-of-Flight) laser ranging module has a specified accuracy of ±1% of full scale (±8 cm at 800 mm), introducing non-negligible distance measurement error that propagates into trajectory calculation. (3) Crosswind effects: Uncontrolled air currents in the laboratory environment (estimated 0.2–0.5 m/s from HVAC systems) exert transverse forces on the jet, causing lateral deviation proportional to flight time. Mitigation strategies include: (a) extending the polynomial training data beyond 80 cm with finer sampling in the breakup regime, (b) implementing a Kalman filter to fuse multiple distance measurements and reduce ranging noise, and (c) adding a wind speed sensor to enable real-time trajectory compensation.

### 6.4. Ablation Study: Component Contribution Analysis

To quantitatively evaluate the contribution of each key technical component to the overall system performance, a systematic ablation study was conducted. Seven configurations were designed, progressively removing or replacing individual components from the full proposed system. All experiments were performed on the same hardware platform (OpenMV4 H7 Plus + STM32F103C8T6) under identical environmental conditions (indoor, 500 lux, 20 °C). The results are summarized in Table 12.

The most significant impact is observed when removing infrared fusion (Configuration D): the false alarm rate surges from 2.0% to 15.3%, confirming that temperature verification is the most critical component for robustness in complex environments. Conversely, replacing the sixth-degree polynomial with an ideal parabola (Configuration F) does not affect detection metrics but degrades extinguishing precision from ±5 cm to ±12 cm at 60 cm range, demonstrating the necessity of the high-order model for air resistance compensation. Similarly, reverting to standard PID control (Configuration G) increases drop-point deviation by 2.5 cm, validating the benefit of the improved dual-loop controller.

### 6.5. Power Consumption and Energy Budget

As an embedded system intended for real-world deployment, the power consumption characteristics of the proposed fire-fighting platform were estimated. The measurement setup employed an INA219 bidirectional current sensor (1 mA resolution, ±0.5% accuracy) connected in series with the power rails of each major subsystem. Measurements were conducted across four operational modes specified by the reviewer: idle (system standby with sensors active), detection (vision inference + IR fusion), tracking (servo-driven fire source locking), and pumping (water discharge + full actuation). The results are presented in Table 13.

In idle mode, the system typically draws approximately 600–700 mW, with the OpenMV4 H7 Plus accounting for the largest share (roughly 450–500 mW) and the combined peripheral sensors (MLX90640, VL53L1X) contributing an additional 30–40 mW. When the detection pipeline is activated, total power rises to approximately 950–1100 mW, as the ARM Cortex-M7 core operates at full load during CNN inference and the ToF sensor enters active ranging. Tracking mode further increases consumption to approximately 1100–1300 mW, driven primarily by the dual-DOF servo pan-tilt during dynamic fire-source positioning. The peak power demand of approximately 5800–6500 mW occurs during full-operation mode, when the 12V high-pressure water pump (approximately 4500–5500 mW) activates simultaneously with all other subsystems. These ranges provide practical guidance for power-budget planning and battery-capacity sizing in field deployments.

Regarding temperature threshold robustness, the default threshold *θ_T_* = 55 °C was selected to exceed maximum ambient temperatures (typically <45 °C in indoor environments) while remaining below the minimum expected fire source temperature (>80 °C for candle flames at 10 cm). To evaluate sensitivity to calibration drift, a tolerance analysis was conducted: varying *θ_T_* by ±5 °C (to 50 °C and 60 °C) changed the false alarm rate by 0.8% and 0.3% respectively on the interference test set, indicating acceptable robustness to moderate sensor calibration uncertainty. Periodic recalibration against a reference blackbody source (emissivity ε = 0.95) is recommended every 6 months per manufacturer specifications.

## 7. Conclusions

Aiming at the prominent problems of high false alarm rate, response lag and insufficient closed-loop capability of traditional fire monitoring systems in complex environments, this study designed and implemented an integrated embedded system for fire source detection, tracking and extinguishing based on multimodal fusion and lightweight models. The system constructs an autonomous closed-loop control architecture of perception–decision–execution–feedback and adopts a heterogeneous computing platform with master–slave coordination of OpenMV4 H7 Plus and STM32C8T6, realizing the full-process intelligent response from fire source perception to precision fire extinguishing in resource-constrained embedded environments. The main conclusions are drawn as follows:

1. A lightweight vision-infrared decision-level fusion recognition mechanism was proposed By fusing visible light images and infrared temperature information based on the Bayesian inference framework, combined with the FOMO model optimized via INT8 quantization and pruning, the system achieves an average recognition accuracy of 95.6% and a false alarm rate of 2.0% on interference-dominant scenarios and an average of 1.4% across all operational conditions, which significantly improves the robustness of the system in complex illumination and interference environments.
2. The established high-precision sixth-order polynomial jet trajectory prediction model fully characterizes the coupled dynamic characteristics of elevation angle, flow rate and air resistance. Within the full parameter range, the goodness of fit R^2^ reaches 0.9975 (adjusted R^2^ = 0.9968) and the root mean square error (RMSE) of drop point prediction is 0.042 m, which provides a reliable theoretical basis for the precise control of fire-extinguishing actuators.
3. For servo control, the introduced error dead zone and output limiting mechanism achieve fast and stable fire source tracking and attitude control. The system tracking adjustment time is 1.25 s, the steady-state error does not exceed 3 pixels, the fire-extinguishing drop point accuracy is better than ±5 cm within the range of 10–60 cm, and the overall system response time is controlled within 8.5 s.
4. Collectively, the embedded intelligent fire protection closed-loop system constructed demonstrates excellent comprehensive performance across a wide illumination range (50–100,000 Lux) and various interference scenarios, with a task success rate of 92%, which verifies its practicability and reliability in real-world applications such as households, warehouses and industrial inspections.

Several practical considerations merit discussion for real-world deployment. The estimated bill-of-materials cost is approximately USD 180, comprising OpenMV4 H7 Plus (USD 85), STM32F103C8T6 (USD 3), thermal sensor MLX90640 (USD 45), ToF ranging module (USD 25), pump and servo actuators (USD 15), and structural components (USD 7). Physical robustness is addressed through IP54-rated enclosure design protecting against water spray and dust ingress; however, direct exposure to temperatures exceeding 70 °C requires additional thermal shielding. The IR-cut filter on the OV5640 (650 nm cutoff) effectively blocks near-infrared radiation from high-temperature backgrounds. System maintenance involves monthly lens cleaning and biannual thermal sensor recalibration against a blackbody reference. Scalability to larger areas can be achieved through multi-unit mesh deployment with centralized coordination, where each unit covers approximately 25 m^2^ at maximum range. Future work will explore integration with building management systems (BMS) via the MQTT protocol for coordinated multi-zone fire suppression.

Building upon the current system, several promising research directions are identified:

1. Advanced sensor fusion: While the current decision-level fusion achieves O(1) complexity, feature-level fusion approaches such as transformer-based cross-modal attention mechanisms could potentially enhance accuracy further on more powerful edge platforms.
2. Expanded fire scenarios: Testing on larger-scale fires (Class A/B fires per NFPA 10) and outdoor environments with uncontrolled wind conditions would validate scalability.
3. Multi-agent coordination: Implementing cooperative fire suppression with multiple robotic units using distributed consensus algorithms to cover extended areas.
4. Moving target tracking: Extending the current static fire source assumption to dynamic fire spread by integrating predictive motion models with the control loop.
5. Adaptive learning: Implementing online model adaptation through federated learning across deployed units to continuously improve detection accuracy without centralized data collection.

Furthermore, while this work prioritizes lightweight decision-level fusion for embedded viability, the emerging landscape of multimodal remote sensing research offers compelling directions for future extension. The heterogeneously salient graph representation of Yang et al. and the language-query transformer architecture of Lan et al. demonstrate that deep feature-level alignment can substantially improve discriminative power in complex scenes. A promising avenue is the development of adaptive multimodal architectures that inherit the low-latency virtues of our Bayesian verification while incorporating selective feature-level refinement modules—e.g., a lightweight cross-modal attention gate activated only when decision-level confidence falls below a threshold. Such hybrid designs could retain the 8.5 s full-cycle response time critical for fire emergencies while approaching the robustness of these more expressive paradigms.

## Figures and Tables

**Table 1:** Comparison of lightweight fire detection models.

Method	Accuracy	Model Size	Speed	Platform	Compression Strategy
Improved YOLOv5s	82.1% mAP@0.5	5.9 MB	79.92 FPS	RTX 3070 (GPU)	None (full precision)
UFS-Net	98.802%	7.99 MB	68 FPS	GTX 1080 (GPU)	Depthwise separable conv
Multi-sensor + CNN	99.1%	-	-	Embedded (unspecified)	Lightweight design
Proposed LFOMO	95.6%	1.8 MB	4.76 FPS	OpenMV4 H7 Plus (480 MHz)	INT8 + Pruning

**Table 2:** Comparison of multimodal fusion strategies.

Method	Fusion Level	Accuracy	Complexity	Latency	Platform
D-S Fusion [7]	Decision-level	0.9818 max prob.	O(n) per node	10–13.5 min	WSN (distributed)
SVM-RCNN [8]	Feature-level	>87%	Hand-crafted features	-	GPU-based
CCMR-PA [9]	Decision-level (GIoU)	F1 = 90.03%	GIoU computation	-	GPU-based
CP-YOLOv11-MF [10]	Mid-term feature (CPCA + PPAS)	96.3% mAP50	O(n2), 11.83M params	-	GPU (23 MB)
LBiF-YOLO [11]	Feature-level (SSAF)	84.93% mAP@0.5	28.9 GFLOPs, 17.29M params	21.2 ms	GPU-based
Proposed LFOMO	Decision-level (Bayesian)	95.6%/2.0% FAR	O(1)	210 ms	OpenMV4 H7 Plus

**Table 3:** Comparison of fire suppression control methods.

Method	Trajectory Model	Precision	Range/Distance	Integration Level	Method
Fire monitor	Geometric aiming	0.10 m pitch error	Long-range	Detection to Actuation (open-loop)	Fire monitor
Euler jet model	Theoretical (Euler)	≤10% flow-rate error	Variable	Model only (no system)	Euler jet model
Coal mine robot	Experimental (K = 0.36)	Range-focused	18.147 m max	Actuation only (no detection)	Coal mine robot
Proposed LFOMO	Sixth-degree polynomial	±5 cm	10–60 cm	Closed-loop	Proposed LFOMO

**Table 4:** Dataset composition overview.

Component	Description	Sample Count	Notes
Self-Collected Data	6 illumination scenarios, 3 fire types, 3 interference types	1000	OV5640 @ 640 × 480, VOC format
Public: FLAME Public: FireNET Public: Kaggle Curated TOTAL	Aerial wildfire imagery, UAV-captured	1500	Screened from 9000+ raw images
	IoT-oriented fire detection images	1000	Screened from 2425 images
	Mixed indoor/outdoor fire scenarios	1000	Screened from Kaggle collections
	9 scene categories, 6 object classes	4500	Train 3600/Test 900 (8:2)

**Table 5:** Fitting statistics.

-	E
Number of Points	132
Degrees of Freedom (DOF)	104
Reduced Chi-Sqr	0.66026
Sum of Squared Residuals (SSR)	68.66714
R-Squared (Coefficient of Determination, COD)	0.99746
Adjusted R-Squared	0.99678

**Table 6:** Analysis of variance (ANOVA).

-	-	DF	Sum of Squares (SS)	Mean Square (MS)	F-Value	Prob > F
Distance	Regression	27	26,918.08987	996.96629	1509.9579	<0.0001
	Residual	104	68.66714	0.66026	-	-
	Uncorrected Total	132	125,912.3421	-	-	-
	Corrected Total	131	26,986.75701	-	-	-

**Table 7:** Optimized PID controller parameters of the pan-tilt.

Controller Type	Proportional Coefficient (*K_p_*)	Integral Coefficient (*K_i_*)	Derivative Coefficient (*K_d_*)	Output Limit
Pan	0.1	0.01 (Enabled when e > 10)	0.012	±8
Tilt	0.09	0.01 (Enabled when e > 10)	0.012	±8

**Table 8:** Optimized PID controller parameters of the fire water cannon.

Controller Type	Proportional Coefficient (*K_p_*)	Integral Coefficient (*K_i_*)	Derivative Coefficient (*K_d_*)	Output Limit
Pan	0.1	0.01 (Enabled when e > 10)	0.01	±8
Tilt	0.15	0.015 (Enabled when e > 10)	0.015	±6

**Table 9:** Experimental scene setup.

Scene Name	Type	
Lighting Scene	Indoor	Indoor Normal Light (500 lux)
		Indoor Low Light (50 lux)
		Indoor Strong Light (5000 lux)
	Outdoor	Sunny Day (100,000 lux)
		Cloudy Day (10,000 lux)
		Nighttime (10 lux)
Fire Scene	Candle Fire (Diameter: 3 cm, Distance: 10–80 cm)	
	Alcohol Fire (Diameter: 5 cm, Distance: 10–80 cm)	
Interference Scene	Candlelight (55 °C)	
	Electric Soldering Iron (60 °C)	
	Sunset Reflection (40 °C)	

**Table 10:** Fire source recognition accuracy across multiple scenarios (table).

Test Scenario	Number of Samples	Number of Correct Recognitions	Accuracy Rate	Number of False Detections	False Detection Rate	Response Time
Indoor Normal Lighting	100	98	98.0%	1	1.0%	260 ms
Indoor Low Light	80	77	96.3%	1	1.3%	250 ms
Outdoor Sunny Day	120	114	95.0%	2	1.7%	280 ms
Outdoor Cloudy Day	100	95	95.0%	1	1.0%	255 ms
Outdoor Nighttime	80	75	93.8%	1	1.3%	240 ms
Interference Scenario	150	-	-	3	2.0%	285 ms
Average	105	92	95.6%	1.5	1.4%	261 ms

**Table 11:** Overall system performance test.

Test Scenario	Number of Successful Tests	Success Rate	Total Time Consumption (s)	Recognition Time Consumption (s)	Tracking Time Consumption (s)	Fire Extinguishing Time Consumption (s)
Indoor Low Light and Interference	46	92%	8.5	0.28	1.25	3.8
Standard Deviation (SD)	-	-	0.5	0.04	0.12	0.3

**Table 12:** Ablation study results.

Config	Configuration Description	Accuracy (%)	Latency (ms)	Model Size (MB)	False Alarm (%)	Ext. Precision (cm)
A	FP32 + No Pruning + IR Fusion + 6th Poly (Non-deployable baseline)	96.2	520	20.8	2.0	±4
B	INT8 + No Pruning + IR Fusion + 6th Poly	95.8	340	5.2	2.0	±4
C	INT8 + Pruning + IR Fusion + 6th Poly (PROPOSED)	95.6	261	1.8	2.0	±5
D	INT8 + Pruning + No IR Fusion + 6th Poly (w/parabola)	86.4	245	1.8	15.3	±5
E	INT8 + No Pruning + IR Fusion + 6th Poly	95.6	280	5.2	2.0	±5
F	INT8 + Pruning + IR Fusion + Ideal Parabola	95.6	261	1.8	2.0	±12
G	INT8 + Pruning + IR Fusion + 6th Poly + Standard PID	95.6	261	1.8	2.0	±7.5

**Table 13:** Power consumption breakdown by hardware module and operational mode.

Hardware Module	Idle Mode	Detection Mode	Tracking Mode	Pumping Mode	Full Operation
OpenMV4 H7 Plus (MCU)	450–500 mW	750–850 mW	700–800 mW	450–500 mW	750–850 mW
STM32F103C8T6 (MCU)	15–25 mW	70–80 mW	70–80 mW	70–80 mW	70–80 mW
MLX90640 IR Sensor	10–20 mW	10–20 mW	10–20 mW	10–20 mW	10–20 mW
VL53L1X ToF Sensor	15–25 mW	15–25 mW	15–25 mW	15–25 mW	15–25 mW
Servo Pan-Tilt (2-DOF)	100–150 mW	100–150 mW	300–400 mW	100–150 mW	300–400 mW
12V Water Pump	<10 mW	<10 mW	<10 mW	4500–5500 mW	4500–5500 mW
TOTAL SYSTEM POWER	600–700 mW	950–1100 mW	1100–1300 mW	5200–5800 mW	5800–6500 mW

## Data Availability

Publicly available datasets were analyzed in this study. This data can be found here: https://www.kaggle.com/datasets/ (accessed on 23 August 2025); https://ieee-dataport.org/open-access/flame-dataset-aerial-imagery-pile-burn-detection-using-drones-uavs (accessed on 23 August 2025); https://github.com/OlafenwaMoses/FireNET (accessed on 23 August 2025) The custom datasets generated during the study are available from the corresponding author on reasonable request.

## Abbreviations

The following abbreviations are used in this manuscript:

FOMO	Faster Objects, More Objects
YOLO	You Only Look Once
CNN	Convolutional Neural Network
ROI	Region Of Interest
